# Biomechanical study of oblique lumbar interbody fusion (OLIF) augmented with different types of instrumentation: a finite element analysis

**DOI:** 10.1186/s13018-022-03143-z

**Published:** 2022-05-14

**Authors:** Xin-Yi Cai, Han-Ming Bian, Chao Chen, Xin-Long Ma, Qiang Yang

**Affiliations:** 1 Department of Spine Surgery, Tianjin Hospital, Tianjin University, 406 Jiefang South Road, Hexi District, Tianjin, 300211 China; 2 Tianjin Key Laboratory for Advanced Mechatronic System Design and Intelligent Control, School of Mechanical Engineering, Tianjin University of Technology, Tianjin, 300384 China; 3 Tianjin Medical University, Tianjin, 300070 China

**Keywords:** Biomechanical study, Lumbar spine, Oblique lumbar interbody fusion, Augmentation, Finite element analysis

## Abstract

**Background:**

To explore the biomechanical differences in oblique lumbar interbody fusion (OLIF) augmented by different types of instrumentation.

**Methods:**

A three-dimensional nonlinear finite element (FE) model of an intact L3-S1 lumbar spine was built and validated. The intact model was modified to develop five OLIF surgery models (Stand-alone OLIF; OLIF with lateral plate fixation [OLIF + LPF]; OLIF with unilateral pedicle screws fixation [OLIF + UPSF]; OLIF with bilateral pedicle screws fixation [OLIF + BPSF]; OLIF with translaminar facet joint fixation + unilateral pedicle screws fixation [OLIF + TFJF + UPSF]) in which the surgical segment was L4–L5. Under a follower load of 500 N, a 7.5-Nm moment was applied to all lumbar spine models to calculate the range of motion (ROM), equivalent stress peak of fixation instruments (ESPFI), equivalent stress peak of cage (ESPC), equivalent stress peak of cortical endplate (ESPCE), and equivalent stress average value of cancellous bone (ESAVCB).

**Results:**

Compared with the intact model, the ROM of the L4–L5 segment in each OLIF surgery model decreased by > 80%. The ROM values of adjacent segments were not significantly different. The ESPFI, ESPC, and ESPCE values of the OLIF + BPSF model were smaller than those of the other OLIF surgery models. The ESAVCB value of the normal lumbar model was less than the ESAVCB values of all OLIF surgical models. In most postures, the ESPFI, ESPCE, and ESAVCB values of the OLIF + LPF model were the largest. The ESPC was higher in the Stand-alone OLIF model than in the other OLIF models. The stresses of several important components of the OLIF + UPSF and OLIF + TFJF + UPSF models were between those of the OLIF + LPF and OLIF + BPSF models.

**Conclusions:**

Our biomechanical FE analysis indicated the greater ability of OLIF + BPSF to retain lumbar stability, resist cage subsidence, and maintain disc height. Therefore, in the augmentation of OLIF, bilateral pedicle screws fixation may be the best approach.

## Highlights

1. OLIF is the popular surgical technique, and its biomechanical research is lacking.
2. Five OLIF surgical models with different fixation instrumentations were developed.
3. The biomechanical differences between these OLIF models were fully analysed.

## Introduction

Degenerative lumbar spine disease is becoming increasingly common and a burden in aging societies [1–4]. Lumbar interbody fusion (LIF) is an effective method for managing degenerative lumbar spine disease. Classical LIFs, including anterior lumbar interbody fusion (ALIF), posterior lumbar interbody fusion (PLIF), and transforaminal lumbar interbody fusion (TLIF), have been used for several decades. Oblique lumbar interbody fusion (OLIF) is a recently developed minimally invasive surgery (MIS) that has been widely adopted worldwide [5–7].

In the OLIF introduced by Mayer in 1997, the lateral space of the spine was reached through the anterior part of the psoas muscle [7]. Later, Silvestre et al. modified the procedure, formally proposing OLIF via MIS, by accessing the disc space through a corridor between the peritoneum and psoas muscle [8]. OLIF has many advantages over other LIF procedures, such as a shorter operation time, less blood loss, a quicker recovery, and a shorter hospital stay [9, 10]. Compared with ALIF, OLIF reaches the lumbar spine via an open window between the anterior vessels and the psoas muscle, thus theoretically avoiding injury to important nearby tissue structures, such as the anterior abdominal large blood vessels and ureters [10, 11]. Compared with PLIF and TLIF, OLIF does not enter the spinal canal or damage posterior structures, such that the risks of nerve root injury and venous plexus bleeding are lower [10]. In addition, OLIF does not pass through the psoas muscle, thereby avoiding injury of the muscle and of the lumbar plexus nerve, unlike in XILF [10, 12].

Clinically, OLIF technique should be supplemented with different internal fixation instrumentations to increase rigidity of the surgical segment [10, 11, 13–18]. Bilateral pedicle screws fixation has the advantage of biomechanical stability as a traditional interbody fusion fixation method, but whose larger rigid fixation may lead to accelerated degeneration of adjacent segments [16, 19]. In recent years, some scholars have tried to use other internal fixation methods (such as OLIF + LPF, OLIF + UPSF, OLIF + UPSF + TFJF) in OLIF surgery, and achieved better effects [16–18]. However, the differences in the biomechanical stability of these internal fixation methods are currently unknown.

Previous OLIF studies have focused largely on clinical outcomes [8, 10, 11, 13], but biomechanics also play an important role in the physiology, pathology, and surgical realignment of the lumbar spine [20–22]. Besides the choice of instrumentation, cage placement is important in achieving improved spinal stability and in reducing the occurrence of cage subsidence after OLIF [23, 24]. However, a clear understanding of both the biomechanics of OLIF and the influence of different fixation systems on the procedure is lacking.

To overcome the difficulty of simulating and testing the physiological behaviours of the lumbar spine in vivo and in vitro, a finite element (FE) method was developed to analyse the biomechanics of the lumbar spine [12, 25–28]. FE analysis not only accurately and conveniently simulates the biomechanical behaviours of spinal structures without damaging them, it also generates biomechanical data that are difficult to obtain either in vivo or in vitro, such as intradiscal pressure, facet joint force, and the stresses on important spinal components. FE models are based on averages and thus better reflect changing trends in data values. Moreover, the repeatability and accuracy of FE are constantly improving, aided by advances in computer simulation software and calibration and validation processes. Thus, in the present study FE analysis was used to investigate the biomechanical differences between Stand-alone OLIF and OLIF augmented with various types of instrumentation.

## Materials and methods

### Development of intact lumbar spine model

**Fig. 1:**
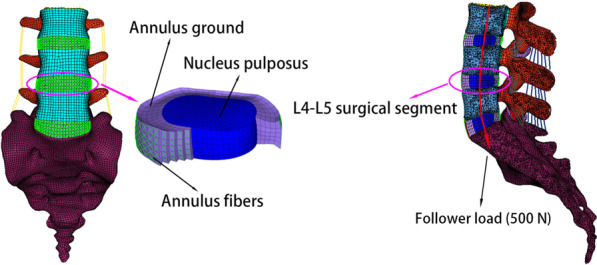
A three-dimensional nonlinear finite element (FE) model of the intact lumbar spine (L3–S1)

A previously validated, three-dimensional, nonlinear FE model of the L3–S1 segment of the whole lumbar spine was used (Fig. 1) [27, 28]. Based on the computed tomography (CT) image of a healthy 30-year-old male (height: 173 cm, weight: 68 kg) in a supine position, the geometric parameters of the FE model of the lumbar spine were obtained. In brief, data from the CT image of the lumbar spine were imported using a medical image control system (Mimics 10.0; Materialise Technologies, Leuven, Belgium) to complete image segmentation of the lower lumbar spine (L3–S1). The geometric model was then reconstructed using reverse engineering software (Geomagic studio 10.0; Geomagic Inc., North Carolina, USA), and the FE meshes of the different spinal components were constructed using computer-aided engineering (CAE) software (Hypermesh 11.0; Altair Engineering Corp, Michigan, USA). Finally, the lumbar spine model was biomechanically simulated using FE analysis software (Abaqus 6.11; Dassault Systemes Simulia Corporation, Pennsylvania, USA).

In this study, the intact lumbar spine FE model was composed of a vertebral body, posterior elements, facet cartilages, intervertebral discs, and ligaments. The vertebral body comprised cortical bone, cancellous bone, and upper and lower endplates. The vertebral body and posterior elements were defined as isotropic, homogeneous elastic materials [25, 29]. The thickness of the cortical bone was 0.5 mm [30, 31]. The facet cartilages and intervertebral discs were modelled using Neo-Hookean and Mooney–Rivlin hyper-elastic materials, respectively [25, 28, 32]. The average initial gap of the facet cartilages was set to 0.1 mm and the interaction between the articular surfaces was defined as frictionless surface-to-surface contact. The intervertebral disc consisted of the nucleus pulposus, annulus ground, and annulus fibres (Fig. 1). The annulus fibres were embedded in annulus ground and their orientation was ± 30° from the horizontal plane [27, 28, 33]. Seven main ligaments were inserted at appropriate positions: anterior longitudinal ligament (ALL), intertransverse ligament (ITL), posterior longitudinal ligament (PLL), capsule ligament (CL), interspinous ligament (ISL), supraspinal ligament (SSL), and flavum ligament (FL) [34]. The annulus fibres and ligaments were developed as spring elements with nonlinear characteristics [27, 35]. The material properties and element types of each component are shown in Table 1 [25, 27–29, 32, 34, 35].

**Table 1:** Material properties and element types in intact model and surgery models of the lumbar spine

Components	Young’s modulus (MPa)	Poisson’s ratio	Element type	References
Cortical bone	12,000	0.3	C3D8R	[25]
Cancellous bone	100	0.4	C3D4	[29]
Cortical endplate	12,000	0.3	C3D8R	[25]
Posterior element	3500	0.25	C3D4	[29]
Sacrum	5000	0.2	C3D4	[27]
Facet cartilage	Neo-Hookean, *C*10 = 2		C3D8RH	[27, 28, 32]
Annulus ground	Mooney–Rivlin, *C*1 = 0.18, *C*2 = 0.045		C3D8RH	[27, 28, 32]
Nucleus pulposus	Mooney–Rivlin, *C*1 = 0.12, *C*2 = 0.03		C3D8RH	[27, 28, 32]
Annulus fibres	Calibrated stress–strain curves		Spring	[27, 35]
Seven ligaments	Calibrated deflection–force curves		Spring	[27, 34, 35]
Cage	3600	0.25	C3D8R	[36, 37]
Screws and rods	110,000	0.28	C3D8R	[36, 37]
Lateral plate	110,000	0.28	C3D8R	[36, 37]

### Development of the surgical lumbar spine model

**Fig. 2:**
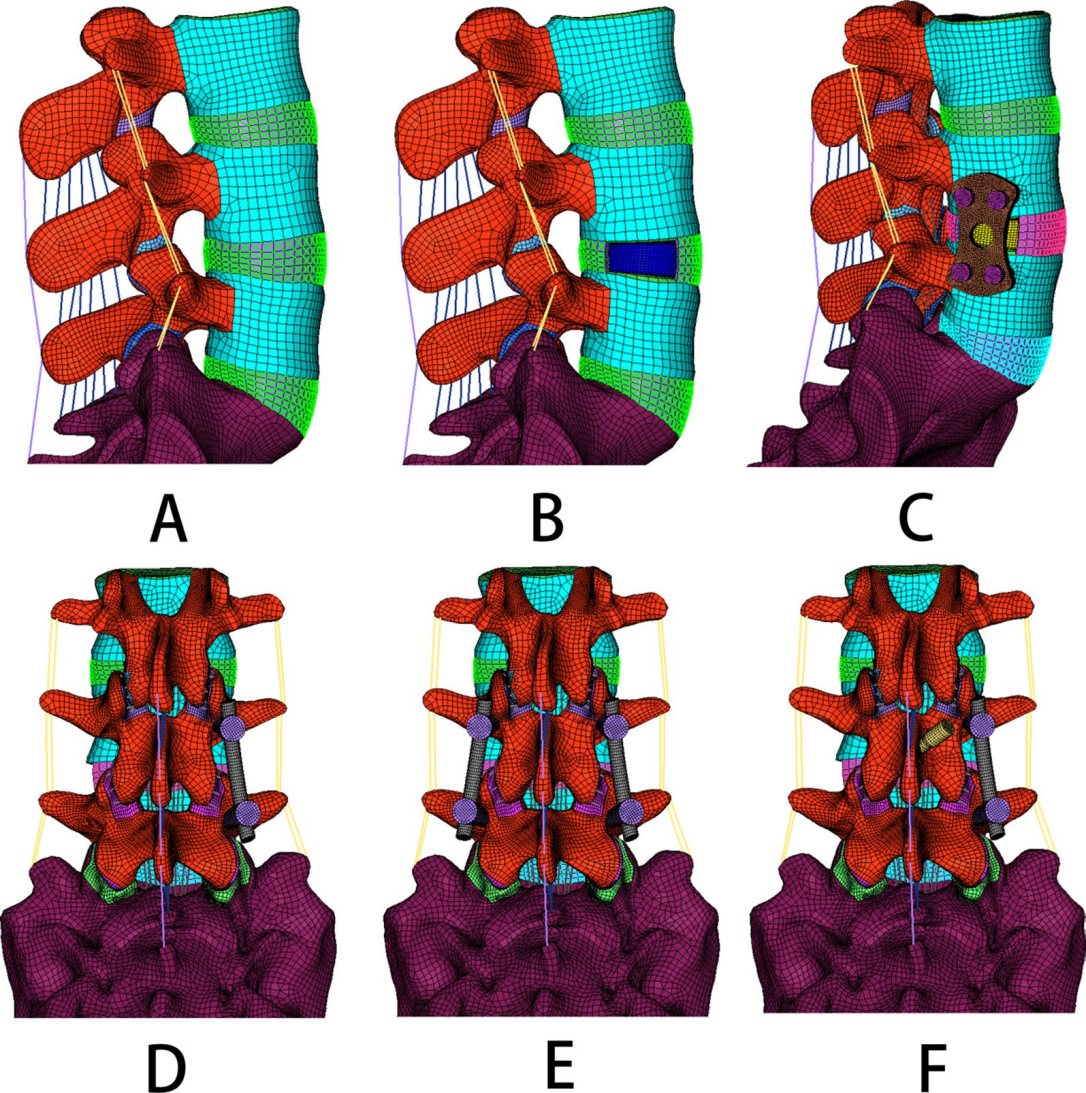
FE models of the lumbar spine (L3–S1). **a** Intact model, **b** Stand-alone oblique lumbar interbody fusion (OLIF), **c** OLIF with lateral plate fixation (OLIF + LPF), **d** OLIF with unilateral pedicle screws fixation (OLIF + UPSF), **e** OLIF with bilateral pedicle screws fixation (OLIF + BPSF), **f** OLIF with translaminar facet joint fixation and unilateral pedicle screws fixation (OLIF + TFJF + UPSF)

This study examined five OLIF surgery models with different fixation instruments: Stand-alone OLIF;OLIF + LPF; OLIF + UPSF; OLIF + BPSF; OLIF + TFJF + UPSF. As the surgical segment, L4–L5 was selected because of its higher incidence of degeneration [25, 38, 39] (Fig. 2b–f).

**Fig. 3:**
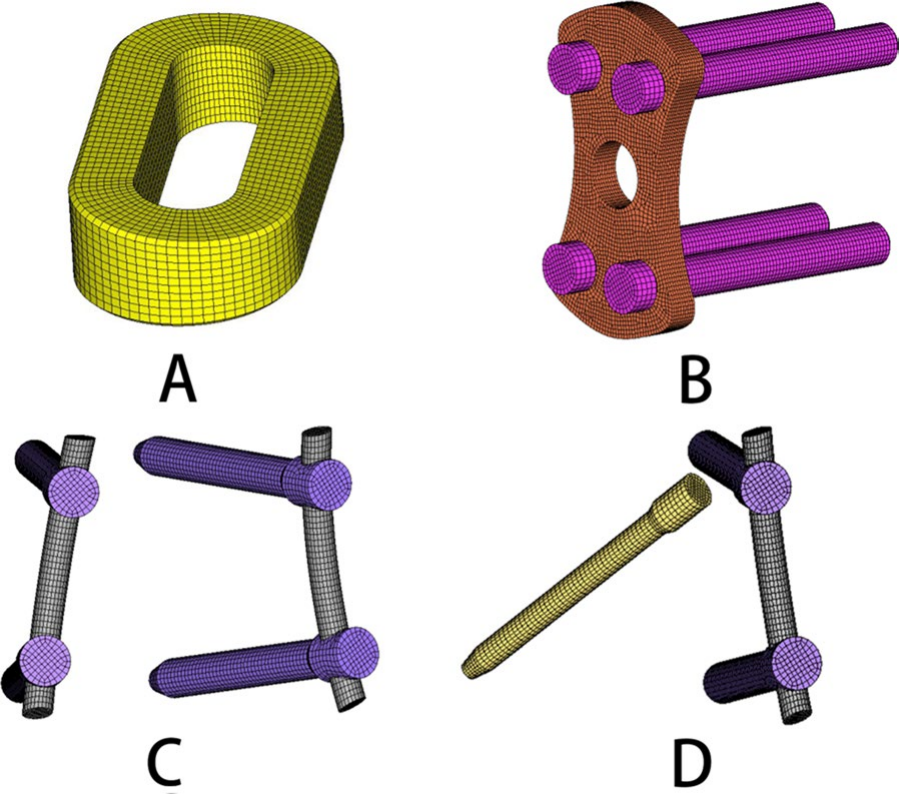
A view of the cage **a** and **b** lateral plate, **c** bilateral pedicle screws, and **d** translaminar facet joint + unilateral pedicle screws fixation instruments

In the CAE software (Hypermesh 11.0), the meshes of the entire nucleus pulposus and part of the annulus were deleted, to simulate their removal during OLIF. In the three-dimensional modelling software (Solidworks 2016; Dassault Systemes Simulia Corporation, Pennsylvania, USA), the OLIF cage and all fixation instruments were established and imported into the Hypermesh software for meshing. The positions of these components were then adjusted to match the lumbar spine model. In other words, to simulate the OLIF surgical procedures with different instrument systems, the entire nucleus pulposus and part of the annulus were removed, and the established OLIF cage (Fig. 3a) was introduced into the damaged intervertebral disc. The different fixation instruments were added at the L4–L5 segment, as shown in Fig. 3b–d. To simplify the OLIF surgical process, the endplate in the OLIF surgical model remained the same as in the normal lumbar spine model.

**Fig. 4:**
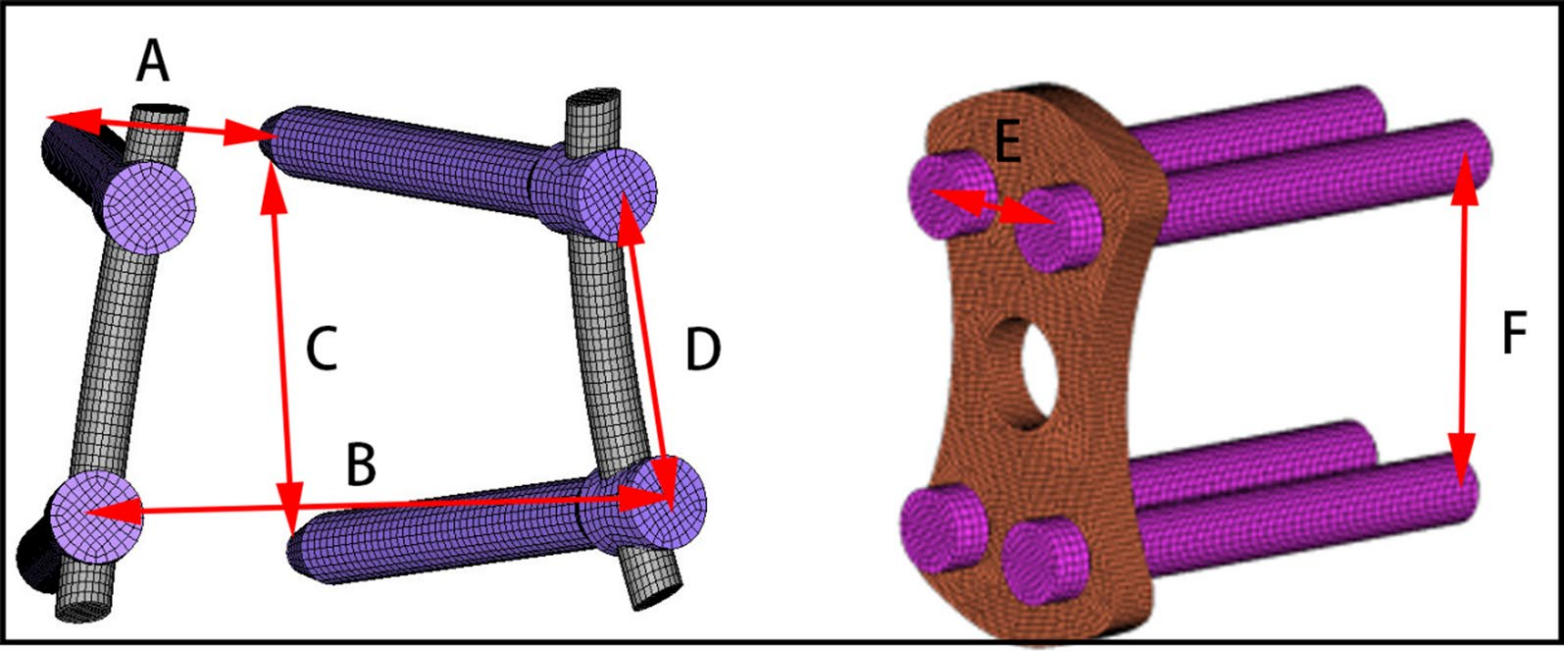
The sizes of the pedicle screws and lateral endplate. **a** Front distances of two upper pedicle screws or two lower pedicle screws; **b** rear distances of two upper pedicle screws; **c** front distance of an upper and a lower pedicle screw; **d** rear distance of an upper and a lower pedicle screw; **e** distances of two upper or two lower screws; **f** distance of the upper and lower pedicle screws

Figure 4 shows a schematic diagram of the distances between some of the fixation instruments. The length of the pedicle screw was 45 mm, and the diameter 6.5 mm. The diameter of the rod was 5.5 mm. The front and rear distances of the two upper or two lower pedicle screws were 19.69 mm and 44.91 mm, respectively. The front and rear distances of the upper pedicle screws and lower pedicle screws were 37.57 mm and 28.37 mm, respectively. The screw–rod and screw–bone interactions were set as a “common nodes” constraint to simulate rigid fixation. The mean length, mean width, height, and surface area of the lateral plate were 40 mm, 18 mm, 3.5 mm, and 22.44 cm^2^, respectively. The distance of the two upper or two lower screws was 11.21 mm. The distance between the upper and lower pedicle screws was 28.01 mm. The interaction of the screw and lateral plate was set as a “Tie” constraint. The length, width, mean height, and surface area of the cage were 45 mm, 22 mm, 9.5 mm, and 28.21 cm^2^, respectively. The interaction of the cage and endplate was also defined as a “Tie” constraint. Note that the “common nodes” and “Tie” interaction have the same effect. The material properties of the pedicle screws, rods, lateral plate, and cage are described in Table 1 [36, 37]. The intact model and the different OLIF surgical models are shown in Fig. 2a–f.

### Loading and boundary conditions

The human lumbar spine is capable of supporting substantial compressive loads in vivo, due to muscle strength and upper body weight. These compressive loads maintain the mechanical stability of the lumbar spine and play an important role in improving its load-carrying capacity [40]. Patwardhan et al. introduced the "follower load" method, in which a compressive preload was applied to a multi-segment lumbar spine specimen without causing its collapse [40]. Based on in vitro experiments, those authors showed that the maximum follower load in the lumbar spine may be as high as 1000 N, but the follower load of the normal lumbar spine is ~ 500 N [40]. Later, a follower load was used in a lumbar spine FE analysis to mimic the substantial compressive in vivo loads arising from muscle strength and upper body weight. The follower load consisted of a physiological compression load along the axis of the lumbar spine (Fig. 1).

In this study, a follower load was applied to the lumbar spine model as described in the literature [27–29]. Specifically, one coupling point was set at the centre of the upper surface of the L3 upper endplate and another coupling point at the centre position of the lower surface of the L3 lower endplate. Similarly, a total of seven coupling points were set in the L3–S1 segment. These coupling points were used to create connector elements. A follower load of 500 N was then applied to each level of the lumbar spine through the connector elements [27–29, 40–42]. Under the follower load (500 N), a moment load of 7.5 Nm was applied to the superior surface of the L3 upper endplate to simulate six different postures (flexion, extension, right bending [RB], left bending [LB], right axial rotation [RAR], and left axial rotation [LAR]). During the loading process, the sacrum was fully constrained to 6 degrees of freedom. The loading and boundary conditions were set using the Hypermesh software.

### Data collection

A von Mises stress is a yield criterion whose value is usually referred to as the equivalent stress. In this study, the biomechanical stability of OLIF achieved with different fixation instruments was assessed based on the range of motion (ROM), the equivalent stress peak of the fixation instruments (ESPFI), the equivalent stress peak of the cage (ESPC), the equivalent stress peak of the cortical endplate (ESPCE), and the equivalent stress average value of cancellous bone (ESAVCB). These biomechanical parameters were calculated using FE analysis software (Abaqus 6.11). ROM refers to the angle of rotation between adjacent lumbar spine segments. The equivalent stress peak and equivalent stress average value were obtained in the post-processing file using the Abaqus software and directly exported. Abaqus is a powerful FE software used in engineering simulation. It has been employed to solve problems ranging from relatively simple linear analyses to complex nonlinear problems. It has also been widely used in FE studies of the spine [12, 26–30, 36].

### Calibration and validation

The calibration process is the preparation stage of the validation process, and the accuracy of the final model depends on the validation results. Thus, the calibration process involves adjusting the correction factors of the ligaments and fibres rather than arbitrarily changing the material properties of other spinal structures. The correction factors (fibres: 0.49; ALL: 1.0; ITL: 1.0; PLL: 0.3; CL: 5.0; ISL: 0.08; SSL: 0.07; FL: 5.0) of the annulus fibres and several ligaments were changed according to the calibration method published by Schmidt et al. [43, 44]. The ROM and disc compression of each segment under different pure moments or a follower compressive load (flexion: 8 Nm, extension: 6 Nm, lateral bending: 6 Nm, axial rotation: 4 Nm; compression: 1200 N) were calculated and the results compared with experimental data from previous studies to validate our FE model of the intact lumbar spine [45]. Boundary and loading conditions were specified and replicated in vitro. All simulations were performed using Abaqus 6.11.

## Results

### Calibration and validation

**Fig. 5:**
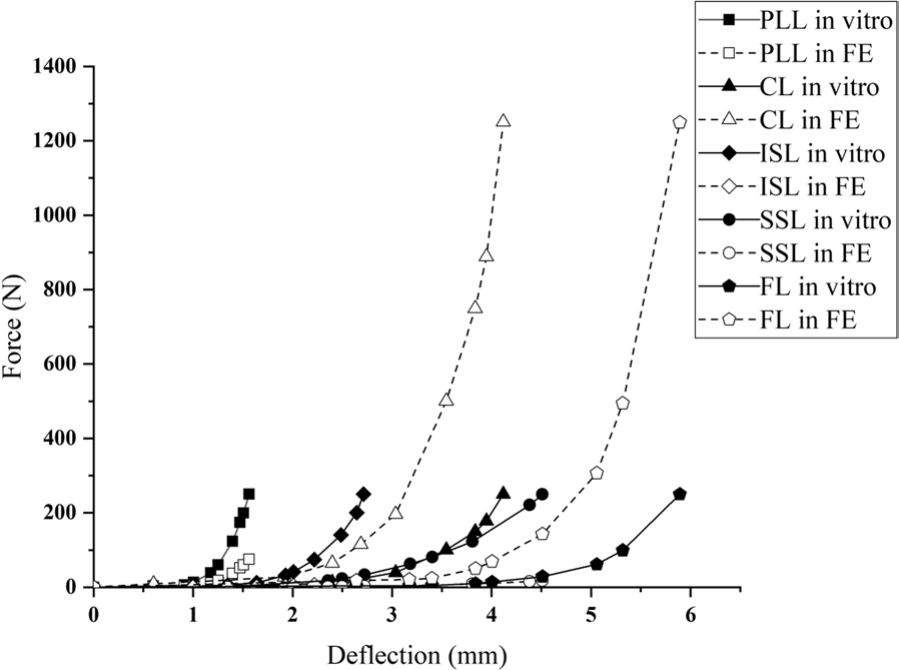
Calibration results of five major ligaments in a model of the intact lumbar spine. *PLL* posterior longitudinal ligament, *CL* capsule ligament, *ISL* interspinous ligament, *SSL* supraspinal ligament, *FL* flavum ligament

**Fig. 6:**
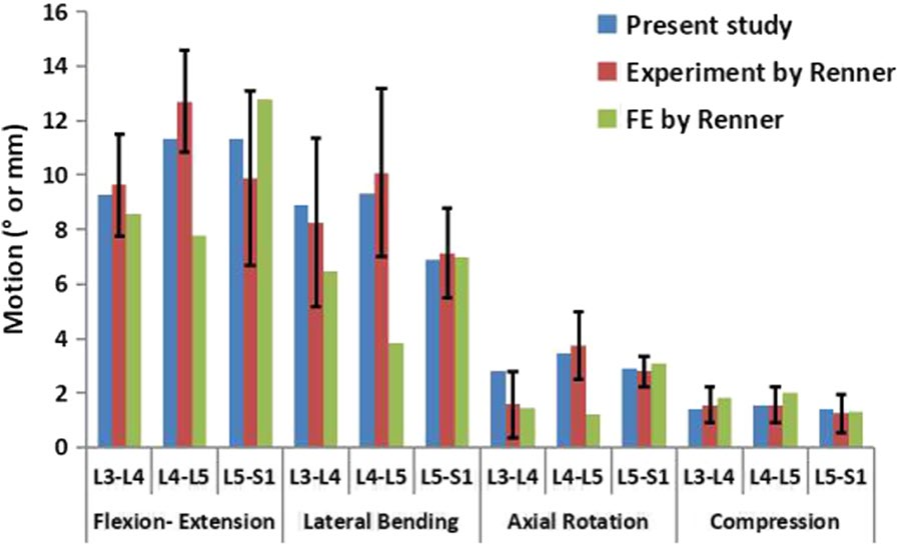
In-vitro and finite element segmental motion values during flexion–extension, lateral bending, axial rotation and compression

Figures 5 and 6 show the calibration and validation results, respectively. Data obtained from our FE model of the intact lumbar spine were compared with the data reported by Renner et al. in their experimental study of lumbar spine motion [45]. The results of our model for all directions (flexion–extension, lateral bending, axial rotation, and compression) were within one standard deviation of those reported by Renner et al. [45]. Therefore, our FE model of the intact lumbar spine was considered to be calibrated and validated, allowing its further use in an analysis of the biomechanical changes of the lumbar spine under different loads.

### Range of motion

**Fig. 7:**
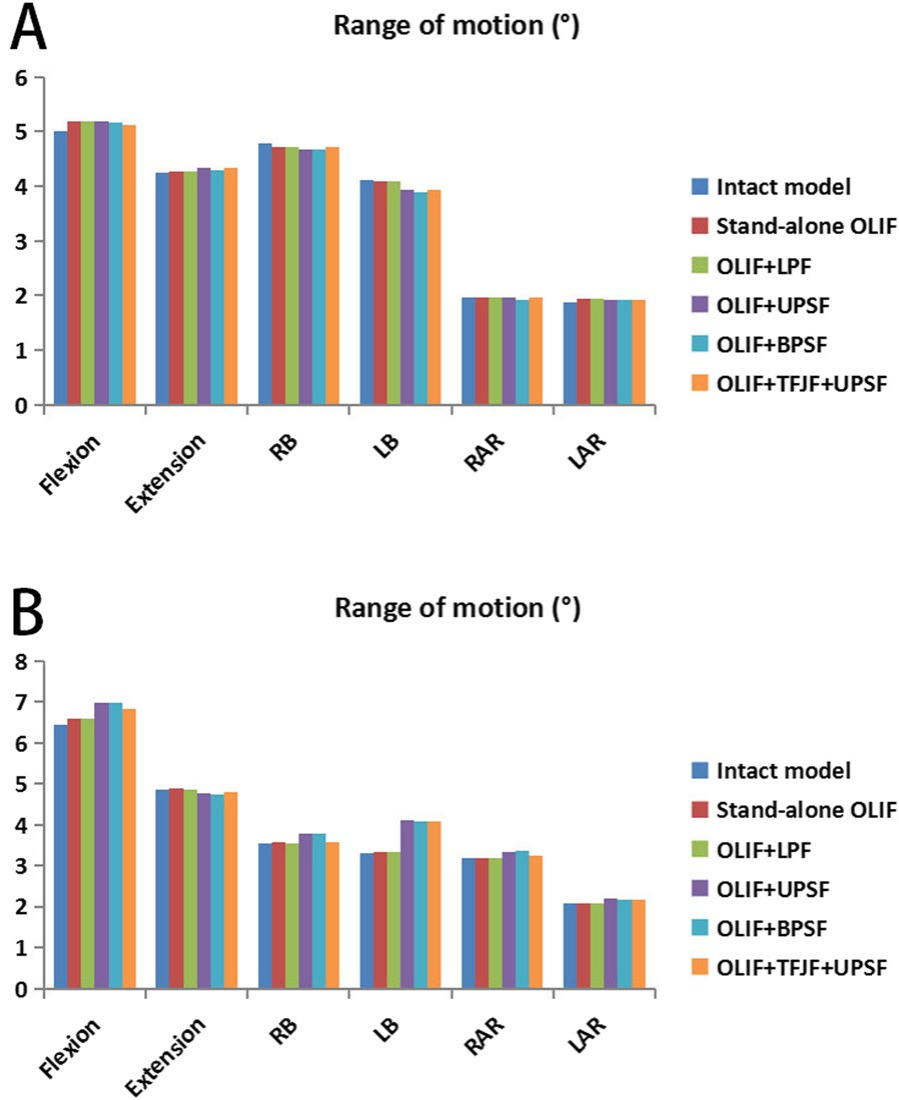
The range of motion at the L3–L4 and L5–S1 segments as obtained with six models and six different postures. **a** L3–L4 segment, **b** L5–S1 segment

**Fig. 8:**
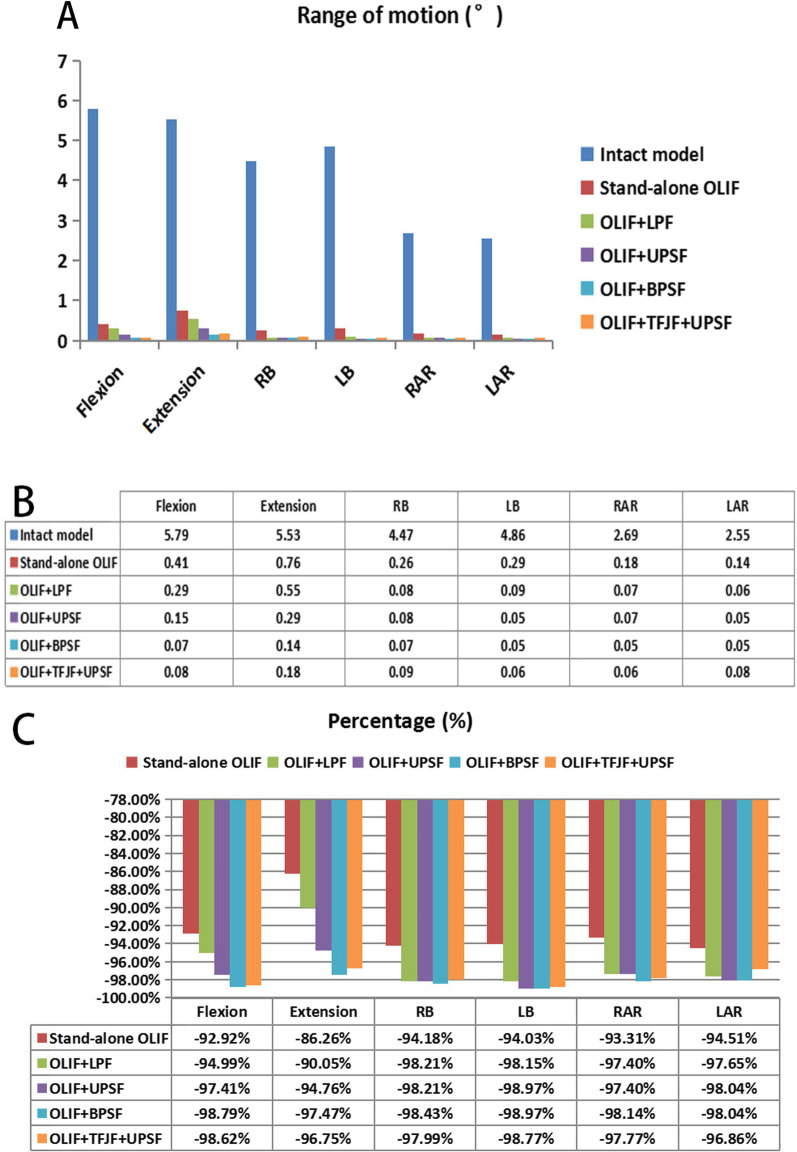
The range of motion (ROM) at the L4–L5 segment and the percentage changes in this segment compared with the intact model for six different postures. **a** L4–L5 segment ROM, **b** Data on the L4–L5 segment ROM, **c** L4–L5 segment-percentage

Figures 7 and 8 show the ROM of segment L3–S1 in six postures both in the intact model and in the five OLIF surgery models. There were no obvious differences in the ROM of segment L3–L4 under the different models (Fig. 7). The ROM of the L5–S1 segment in each surgery model showed a slight increasing trend in all postures (except extension) relative to the intact model, whereas in all of the surgical models the ROM of segment L4–L5 decreased by > 80% (Fig. 8c). In the Stand-alone OLIF model, the ROM of the surgery segment in flexion, extension, RB, LB, RAR, and LAR was reduced by 92.92%, 86.26%, 94.18%, 94.03%, 93.31%, and 94.51%, respectively, compared with the intact lumbar spine model. The ROM of the surgery segment in each posture was larger in the Stand-alone OLIF model than in the other OLIF surgery models. The ROM of the surgery segment was smaller in the OLIF + BPSF model than in the other OLIF models. The differences in the ROM values of the surgery segment in the OLIF + LPF, OLIF + UPSF, and OLIF + TFJF + UPSF models were not significant (Fig. 8a, b). The percent change in the ROM of the L4–L5 surgical segment versus the L4–L5 segment in the intact model is shown in Fig. 8c.

### Equivalent stress peak of fixation instruments

**Fig. 9:**
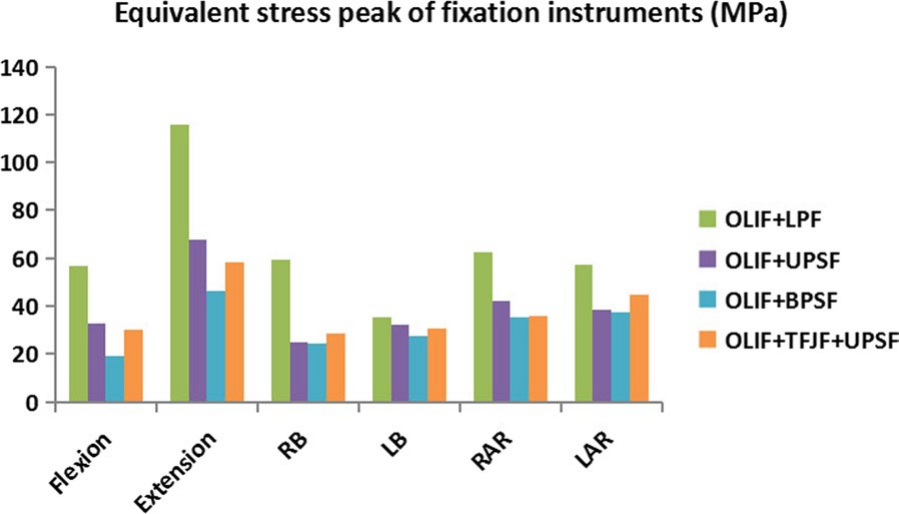
Equivalent stress peak of the fixation instruments as determined in four OLIF models (OLIF + LPF, OLIF + UPSF, OLIF + BPSF and OLIF + TFJF + UPSF) and for six postures

The ESPFI values under the different postures in the four surgery models are shown in Fig. 9. The ESPFI was smaller in the OLIF + BPSF model (18.94–46.35 MPa) than in the other models. The OLIF + LPF model had a much larger ESPFI than did the other OLIF models, with the values in the latter ranging from 35.27 to 116.01 MPa. The ESPFI of the OLIF + UPSF model was larger than that of the OLIF + TFJF + UPSF model in flexion and extension, but the ESPFI values of the two models in the other postures were similar, ranging from 32.39 to 67.92 MPa and from 29.88 to 58.11 MPa, respectively. The ESPFI of the OLIF + UPSF model was slightly larger than that of the OLIF + TFJF + UPSF model.

### Equivalent stress peak of the cage

**Fig. 10:**
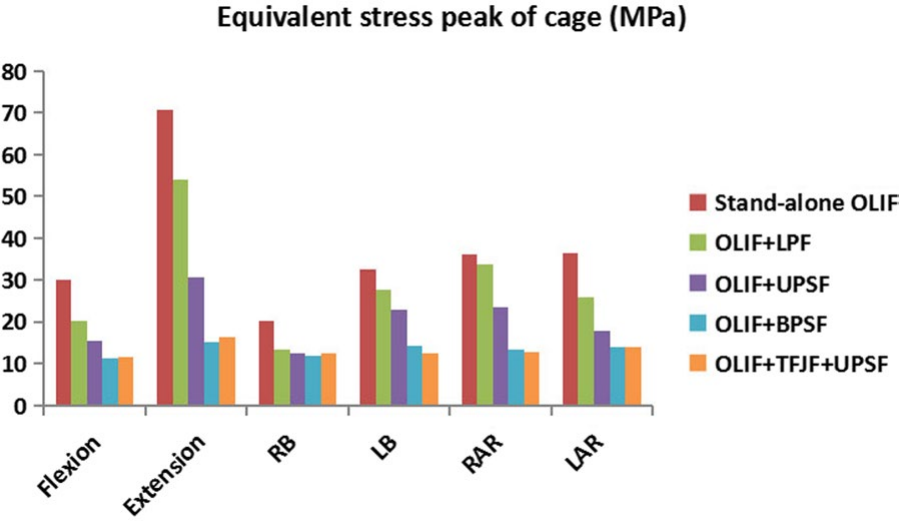
Equivalent stress peak of the cage as determined in five OLIF models (Stand-alone OLF, OLIF + LPF, OLIF + UPSF, OLIF + BPSF and OLIF + TFJF + UPSF) and for six postures

The ESPC values of the five surgery models under different motion states are provided in Fig. 10. The ESPC was larger in the Stand-alone OLIF model than in the other surgery models at each posture. The ESPC values of the OLIF + LPF and OLIF + UPSF models were higher than those of the OLIF + BPSF and OLIF + TFJF + UPSF models, while the ESPC of the OLIF + LPF model was larger than that of the OLIF + UPSF model. The ESPC values of the OLIF + BPSF and OLIF + TFJF + UPSF models were generally similar. Among the models, the OLIF + BPSF model had the smallest ESPC. The ESPC values of the Stand-alone OLIF, OLIF + LPF, OLIF + UPSF, OLIF + BPSF, and OLIF + TFJF + UPSF models were 20.13–70.51 MPa, 13.41–33.49 MPa, 12.41–30.58 MPa, 11.18–14.96 MPa, and 11.65–16.41 MPa, respectively.

### Equivalent stress peak of the cortical endplate

**Fig. 11:**
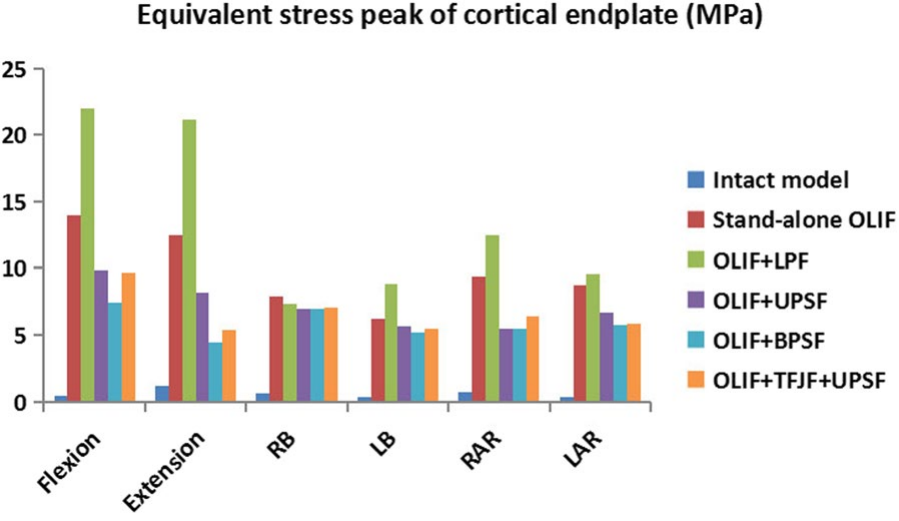
Equivalent stress peak of the cortical endplate as determined in the intact model and in five OLIF models (Stand-alone OLF, OLIF + LPF, OLIF + UPSF, OLIF + BPSF, and OLIF + TFJF + UPSF) for six postures

The ESPCE values of the L4–L5 segment of the intact model and the five OLIF surgery models under different postures are shown in Fig. 11. The ESPCE of the intact model was much smaller than the corresponding values of the five OLIF surgery models. In the OLIF surgery models, the smallest ESPCE was that of the OLIF + BPSF model, whereas the ESPCE values of the Stand-alone OLIF and OLIF + LPF models were larger than those of the OLIF + UPSF, OLIF + BPSF, and OLIF + TFJF + UPSF models. The ESPCE of the OLIF + LPF model was larger than that of the Stand-alone OLIF model. In flexion and extension, the ESPCE of the OLIF + UPSF model was larger than that of either the OLIF + BPSF or the OLIF + TFJF + UPSF model. In other postures, the ESPCE of these three OLIF surgery models did not obviously differ. The ESPCE values in the intact, Stand-alone OLIF, OLIF + LPF, OLIF + UPSF, OLIF + BPSF, and OLIF + TFJF + UPSF models were in the range of 0.35–1.15 MPa, 7.91–13.93 MPa, 7.35–22.02 MPa, 5.51–9.82 MPa, 4.41–7.42 MPa, and 5.35–9.68 MPa, respectively.

### Equivalent stress average value of cancellous bone

**Fig. 12:**
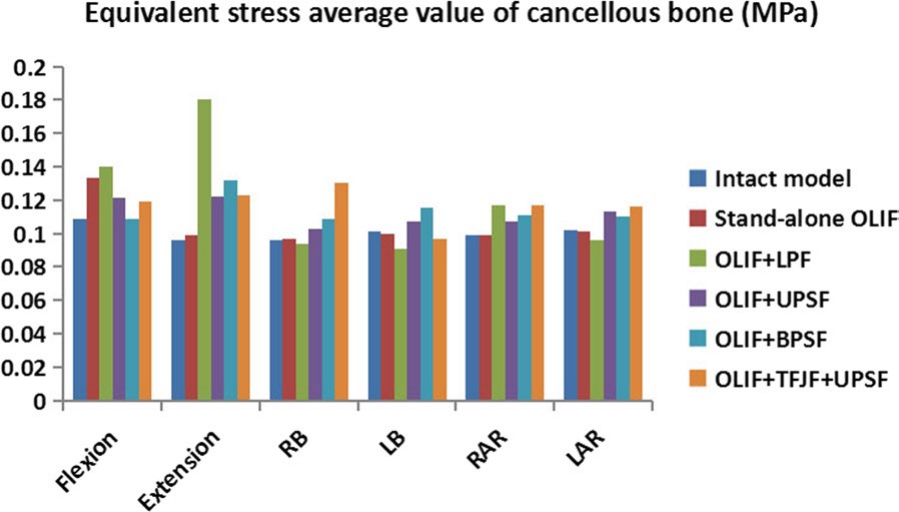
Equivalent stress average value of cancellous bone as determined in the intact model and in five OLIF models (Stand-alone OLF, OLIF + LPF, OLIF + UPSF, OLIF + BPSF and OLIF + TFJF + UPSF) for six postures

The ESAVCB values of L5 in the intact and five OLIF surgery models under different motion states are shown in Fig. 12. The average values were calculated to eliminate the stress concentration caused by the holes in cancellous bone, thus avoiding the abnormal stress caused by stress concentration and allowing the change trend in the ESAVCB to be more accurately determined. The ESAVCB values of the intact and Stand-alone OLIF model did not obviously differ except in flexion. In the OLIF + LPF model, the ESAVCB was much higher than in the other models in flexion–extension, whereas in the other postures the values were generally similar. The ESAVCB values of the OLIF + UPSF, OLIF + BPSF, and OLIF + TFJF + UPSF models were larger than those of the intact model in all postures. The ESAVCB values in the intact, Stand-alone OLIF, OLIF + LPF, OLIF + UPSF, OLIF + BPSF, and OLIF + TFJF + UPSF models were 0.096–0.109 MPa, 0.097–0.133 MPa, 0.091–0.181 MPa, 0.103–0.122 MPa, 0.109–0.132 MPa, and 0.097–0.131 MPa, respectively.

## Discussion

In this study, five important biomechanical parameters (ROM, ESPC, ESPFI, ESPCE, and ESAVCB) were measured to determine the biomechanical differences of OLIF augmented by different instrumentations. ROM and EPSFI reflected the stability of lumbar spine after spinal fusion, as demonstrated in previous studies [19, 36, 37, 46–49], other biomechanical parameters (ESPC, ESPCE and ESAVCB) represented the stress environment of intervertebral disc, the resistance of cage subsidence and disc height maintenance, respectively [36, 37, 50–52].

The stability of surgical segment is a crucial indicator of successful LIF surgery, as instability may be accompanied by complications such as cage subsidence and non-fusion, resulting in pain and possibly work disability for the patient [8, 10]. In all postures, the ROM of the surgical segment in the five OLIF surgery models decreased by > 80% (86.26–98.97%) compared with the intact model, consistent with the trend reported in previous studies [36, 37, 46–48]. Lu et al. [36] explored the biomechanical performances of four types of LIF surgery (PLIF, TLIF, XLIF, and OLIF). The ROM of the surgical segment was reduced by 75.3–92.6% when surgery was combined with bilateral pedicle screws fixation. The ROM of the surgical segment in their OLIF model decreased by > 80% (86.9–89.7%) in all directions [36]. Chen et al. [37] developed single-segment crenel lateral interbody fusion (CLIF) surgery models and showed that the respective ROM values decreased by 76.84–97.97% compared with the normal model. Oxland et al. [46] published a literature review and they found the maximum reduction of the ROM at the index level was 90%, achieved using LIF procedures. Hector et al. used cadaver specimens to simulate OLIF and direct lateral interbody fusion (DLIF) procedures while testing intersegmental rotation [47]. They found that the addition of posterior instrumentations (bilateral pedicle screws) to the interbody spacer increased the stability of the construct significantly, regardless of cage insertion trajectory or screws type. These studies demonstrated that OLIF surgery can provide good stability for the surgical segment.

Our results showed that the ROM was larger in the Stand-alone OLIF model than in the other four OLIF surgical models, and the OLIF + BPSF model had the smallest ROM. And the smaller ROM reflects better segmental stability in OLIF model to a certain extent. These results demonstrated the different impacts of the different instrumentation systems with respect to the stability of the surgery segment, as they increased the stiffness of the surgical segment to different degrees. The larger the increase in the stiffness of the surgical segment, the smaller in the ROM of that segment. Lai et al. conducted a cadaveric study of multilevel lateral lumbar interbody fusion (LLIF) and they found that, even in multi-segment LLIF surgery, the bilateral pedicle screws fixation system provided greater stability than that obtained with other fixation systems (unilateral pedicle screws and lateral plate) [48]. Since the stability of the spine as a whole hinders excessive deviation of each spinal motion segment, the middle area between the vertebrae is kept within the physiological limit. The results of our FE study matched those of in vitro cadaver experiments [36, 37, 46–48], which indicated that bilateral pedicle screws fixation for OLIF could provide greater stability than other fixation instruments.

The changes in the ROM of the segments adjacent to the L4–L5 surgical segment in OLIF models were very small compared with the intact model (Fig. 7). From a biomechanics point of view, fusion surgery unites an originally movable joint, which greatly increases the rigidity of the surgical segment. Compensatory efforts to maintain the overall ROM of the spine may decrease the stiffness and increase the ROM of other segments. However, this scenario was not the case in our study, perhaps due to its simulation of the timely effect of spinal fusion, as our FE simulation represented the immediate post-operative period and a change in the ROM of adjacent segments is a progressive process that may not appear in the early postoperative stage. In previous studies [19, 29] we showed that both disc degeneration and OLIF can cause the degeneration of adjacent segments. Wang et al. compared the ROM of adjacent segments after OLIF and TLIF and found that the two surgeries similarly increased the potential risk of adjacent segment degeneration [49]. Therefore, our model can be improved, with the use of more advanced method needed to simulate this progressive change.

The ESPFI in the OLIF + LPF model was significantly higher than other models (Fig. 9). Chen et al. conducted a FE study on CLIF surgery and they found that the peak stress of lateral plate fixation instrument was larger than other fixation systems, the peak stresses of lateral plate were between 39.6 and 145.8 MPa in different postures [37]. This range is slightly larger than that determined in our study (35.27–116.01 MPa), most likely because, in the former, only the L4–L5 segment was evaluated. In contrast, we constructed an L3–S1 segment model, which allowed stresses of the surgical segment to be assigned to adjacent segments. The greater stress loading observed in the lateral plate could result in instrumentation failure, which would explain why in clinical practice the lateral plate is not often used as an independent fixation system, especially for patients with osteoporosis [37, 50–52]. For this reason, the lateral plate is mostly used for supplementary fixation in combination with other instruments [10, 53]. Among the surgical models tested in our study, the OLIF + BPSF model had the smallest ESPFI. Bilateral pedicle screws provide strong fixation for the surgical segment, as they result in sharing of most of the load from the cephalad direction and significantly decrease the load transferred to the anterior column [36]. Thus, in terms of a reduction in the peak stress of the fixation instrument, a screw–rod may be better than a screw–plate. From this perspective, the BPSF system provides the most stability and LPF the least stability in OLIF, which would account for the prevalence of BPSF systems in clinical practice [54].

The ESPC was larger in Stand-alone OLIF model than in the other OLIF models (Fig. 10). The larger ESPC may damage the adjacent endplates, leading to an abnormal increase in endplate stress and possibly also to degeneration. Destruction of the endplate’s physiological environment increases the risk of cage subsidence and disc space collapse [23, 24]. Cage subsidence is a frequent complication in LIF surgery, and the reduction in disc height is often associated with adjacent segment degeneration [8, 10, 19, 23, 25]. Once the cage subsides and the disc height decreases, the endplate and cancellous bone in the surgical segment may be further damaged, which may lead to changes in the biomechanics of adjacent segments [36]. At the same time, previous in vitro and FE studies demonstrated that the ESPC values represented to some extent the ability of resisting cage subsidence and maintaining disc height [37, 47–49]. In this study, the ESPC values of the OLIF + BPSF and OLIF + TFJF + UPSF models were similar and much smaller than the values of the other surgical models. Thus, in these two models, the ability to resist cage subsidence and maintain disc height was greater. In addition, in terms of restricting segment motion and preventing instrumentation failure, the OLIF + BPSF and OLIF + TFJF + UPSF models had smaller ROM and ESPFI values, indicative of greater stability. Combining the above three biomechanical parameters (ROM, ESPFI and ESPC), OLIF + BPSF model can thus be expected to provide the strongest resistance to cage subsidence and to maintain disc height.

The ESPCE of each OLIF surgical model was much higher than the ESPCE of intact model (Fig. 11). Excessive peak stress may cause damage and rupture of the endplate or even risk accelerating endplate degeneration, which in turn could accelerate disc degeneration [55]. Because the endplate is a bridge for transferring nutrients, endplate degeneration may also interrupt nutrient transport, impairing the vitality of intervertebral disc cells [56]. Therefore, cage design should be improved to increase the contact area between the cage and the endplate, to reduce endplate stress after OLIF surgery. A better understanding of the changes in the endplate stress of the surgical segment after OLIF surgery, and therefore improved clinical treatment, requires further analysis of the mechanism underlying lumbar degenerative diseases.

The ESAVCB in the Stand-alone OLIF model was very close to that of the intact model (Fig. 12), perhaps because the micro-environment of cancellous bone stress in the Stand-alone model was similar to that of the intact model. At the same time, all of the fixation systems provided strong support for OLIF models, such that the ESAVCB values obtained in the OLIF models with fixation instrumentations were higher than the ESAVCB of the intact model. In the different OLIF models of this study, the stress on the proximal fixation system increased, causing it to concentrate on the contact interface between the cancellous bone and the fixation instrument. We therefore measured the equivalent stress average value of cancellous bone to avoid both stress concentration and inaccurate the change trends of ESAVCB. A previous study that analysed the biomechanical performance of different lumbar interbody fusion surgeries found that larger cancellous and endplate stress peaks can lead to cage subsidence and a reduction of disc height [36]. In summary, in most postures the ESAVCB and ESPCE were largest in the OLIF + LPF model and smallest in the OLIF + BPSF model. Thus, compared to the other models, cage subsidence resistance and disc height maintenance may be better in the OLIF + BPSF model and worse in the OLIF + LPF model.

There are several limitations in our study. Firstly, the paravertebral muscles were not simulated in our FE model, which may have misrepresented the true motion of the lumbar spine and the stress distribution of some spinal components. Secondly, the ligaments were simulated as one-dimensional nonlinear spring elements, as their complicated actual structure and the difficulty in reproducing three-dimensional structures hindered their more accurate representation. Both of these limitations affected the FE analysis of the lumbar spine. Thirdly, the geometric morphology of the lumbar spine, including disc height, disc degeneration, and facet joint degeneration, varies from person to person. Our intact model was developed based on the geometric information of the lumbar spine obtained from a single person. Therefore, to a certain extent, our model could only reflect the changes in the biomechanical trends of the lumbar spine under different loads. Finally, relying solely on the ROM as the validation target is a common defect in spinal research using FE models. Nonetheless, despite these limitations and simplifications, the data obtained with our FE model of the lumbar spine showed good consistency with published experimental data and can be used to explore the biomechanical effects of different instruments in OLIF surgery.

## Conclusions

By comparing biomechanical parameters (ROM and ESPFI), our study showed the better stability achieved with the OLIF + BPSF model than with the other OLIF models. In comparisons of biomechanical parameters (ESPC, ESPCE, and ESAVCB), this model also provided the greatest cage subsidence resistance and disc height maintenance. Based on these findings, BPSF offers optimal augmentation for OLIF surgery.

## Data Availability

The datasets used and/or analysed during the current study are available from the corresponding author on reasonable request.

## Abbreviations

LIF: Lumbar interbody fusion
ALIF: Anterior lumbar interbody fusion
PLIF: Posterior lumbar interbody fusion
TLIF: Transforaminal lumbar interbody fusion
XLIF: Extreme lumbar interbody fusion
OLIF: Oblique lumbar interbody fusion
MIS: Minimally invasive surgery
FE: Finite element
OLIF + LPF: OLIF with lateral plate fixation
OLIF + UPSF: OLIF with unilateral pedicle screws fixation
OLIF + BPSF: OLIF with bilateral pedicle screws fixation
OLIF + TFJF + UPSF: OLIF with translaminar facet joint fixation + unilateral pedicle screws fixation
ROM: Range of motion
ESPFI: Equivalent stress peak of fixation instruments
ESPC: Equivalent stress peak of cage
ESPCE: Equivalent stress peak of cortical endplate
ESAVCB: Equivalent stress average value of cancellous bone
ALL: Anterior longitudinal ligament
ITL: Intertransverse ligament
PLL: Posterior longitudinal ligament
CL: Capsule ligament
ISL: Interspinous ligament
SSL: Supraspinal ligament
FL: Flavum ligament
RB: Right bending
LB: Left bending
RAR: Right axial rotation
LAR: Left axial rotation
CLIF: Crenel lateral interbody fusion
DLIF: Direct lateral interbody fusion

## Declarations

### Statement

All methods were carried out in accordance with relevant guidelines and regulations. This paper did not include experimental protocols, so the approval of a named institution or licensing committee was not applicable.

## References

[1] Yorimitsu E, Chiba K, Toyama Y, Hirabayashi K (2001). Long-term outcomes of standard discectomy for lumbar disc herniation: a follow-up study of more than 10 years. Spine.

[2] Walker BF (2000). The prevalence of low back pain: a systematic review of the literature from 1966 to 1998. J Spinal Disord.

[3] Machado GC, Maher CG, Ferreira PH, Harris IA, Deyo RA, McKay D, Li Q, Ferreira ML (2017). Trends, complications, and costs for hospital admission and surgery for lumbar spinal stenosis. Spine.

[4] Von Forell GA, Stephens TK, Samartzis D, Bowden AE (2015). Low back pain: a biomechanical rationale based on "patterns" of disc degeneration. Spine.

[5] Ozgur BM, Agarwal V, Nail E, Pimenta L (2010). Two-year clinical and radiographic success of minimally invasive lateral transpsoas approach for the treatment of degenerative lumbar conditions. SAS J.

[6] Anand N, Baron EM, Khandehroo B, Kahwaty S (2013). Long-term 2- to 5-year clinical and functional outcomes of minimally invasive surgery for adult scoliosis. Spine.

[7] Mayer HM (1997). A new microsurgical technique for minimally invasive anterior lumbar interbody fusion. Spine.

[8] Silvestre C, Mac-Thiong JM, Hilmi R, Roussouly P (2012). Complications and morbidities of mini-open anterior retroperitoneal lumbar interbody fusion: oblique lumbar interbody fusion in 179 patients. Asian Spine J.

[9] Kalakoti P, Missios S, Maiti T, Konar S, Bir S, Bollam P, Nanda A (2016). Inpatient outcomes and postoperative complications after primary versus revision lumbar spinal fusion surgeries for degenerative lumbar disc disease: a national (nationwide) inpatient sample analysis, 2002–2011. World Neurosurg.

[10] Mobbs RJ, Phan K, Malham G, Seex K, Rao PJ (2015). Lumbar interbody fusion: techniques, indications and comparison of interbody fusion options including PLIF, TLIF, MI-TLIF, OLIF/ATP, LLIF and ALIF. J Spine Surg (Hong Kong).

[11] Quillo-Olvera J, Lin GX, Jo HJ, Kim JS (2018). Complications on minimally invasive oblique lumbar interbody fusion at L2–L5 levels: a review of the literature and surgical strategies. Ann Transl Med.

[12] Liu X, Ma J, Park P, Huang X, Xie N, Ye X (2017). Biomechanical comparison of multilevel lateral interbody fusion with and without supplementary instrumentation: a three-dimensional finite element study. BMC Musculoskelet Disord.

[13] Li JX, Phan K, Mobbs R (2017). Oblique lumbar interbody fusion: technical aspects, operative outcomes, and complications. World Neurosurg.

[14] Allain J, Dufour T (2020). Anterior lumbar fusion techniques: ALIF, OLIF, DLIF, LLIF, IXLIF. Orthop Traumatol Surg Res OTSR.

[15] Huo Y, Yang D, Ma L, Wang H, Ding W, Yang S (2020). Oblique lumbar interbody fusion with stand-alone cages for the treatment of degenerative lumbar spondylolisthesis: a retrospective study with 1-year follow-up. Pain Res Manag.

[16] Wen J, Shi C, Yu L, Wang S, Xi Y, Ye X (2020). Unilateral versus bilateral percutaneous pedicle screw fixation in oblique lumbar interbody fusion. World Neurosurg.

[17] Ge T, Ao J, Li G, Lang Z, Sun Y (2021). Additional lateral plate fixation has no effect to prevent cage subsidence in oblique lumbar interbody fusion. J Orthop Surg Res.

[18] Huang P, Wang Y, Xu J, Xiao B, Liu J, Che L, Mao K (2017). Minimally invasive unilateral pedicle screws and a translaminar facet screw fixation and interbody fusion for treatment of single-segment lower lumbar vertebral disease: surgical technique and preliminary clinical results. J Orthop Surg Res.

[19] Du CF, Cai XY, Gui W, Sun MS, Liu ZX, Liu CJ, Zhang CQ, Huang YP (2021). Does oblique lumbar interbody fusion promote adjacent degeneration in degenerative disc disease: a finite element analysis. Comput Biol Med.

[20] Lomelí-Rivas A, Larrinúa-Betancourt JE (2019). Biomechanics of the lumbar spine: a clinical approach. Acta Ortop Mex.

[21] Wilder DG, Pope MH, Frymoyer JW (1988). The biomechanics of lumbar disc herniation and the effect of overload and instability. J Spinal Disord.

[22] Stemper BD, Baisden JL, Yoganandan N, Shender BS, Maiman DJ (2014). Mechanical yield of the lumbar annulus: a possible contributor to instability: laboratory investigation. J Neurosurg Spine.

[23] Le TV, Baaj AA, Dakwar E, Burkett CJ, Murray G, Smith DA, Uribe JS (2012). Subsidence of polyetheretherketone intervertebral cages in minimally invasive lateral retroperitoneal transpsoas lumbar interbody fusion. Spine.

[24] Palepu V, Helgeson M, Molyneaux-Francis M, Nagaraja S (2018). The effects of bone microstructure on subsidence risk for ALIF, LLIF, PLIF, and TLIF spine cages. J Biomech Eng.

[25] Ruberté LM, Natarajan RN, Andersson GB (2009). Influence of single-level lumbar degenerative disc disease on the behavior of the adjacent segments–a finite element model study. J Biomech.

[26] Ambati DV, Wright EK, Lehman RA, Kang DG, Wagner SC, Dmitriev AE (2015). Bilateral pedicle screw fixation provides superior biomechanical stability in transforaminal lumbar interbody fusion: a finite element study. Spine J Off J N Am Spine Soc.

[27] Du CF, Yang N, Guo JC, Huang YP, Zhang C (2016). Biomechanical response of lumbar facet joints under follower preload: a finite element study. BMC Musculoskelet Disord.

[28] Du C, Mo Z, Tian S, Wang L, Fan J, Liu S, Fan Y (2014). Biomechanical investigation of thoracolumbar spine in different postures during ejection using a combined finite element and multi-body approach. Int J Numer Methods Biomed Eng.

[29] Cai XY, Sun MS, Huang YP, Liu ZX, Liu CJ, Du CF, Yang Q (2020). Biomechanical effect of L(4) -L(5) intervertebral disc degeneration on the lower lumbar spine: a finite element study. Orthop Surg.

[30] Cai XY, Sang D, Yuchi CX, Cui W, Zhang C, Du CF, Liu B (2020). Using finite element analysis to determine effects of the motion loading method on facet joint forces after cervical disc degeneration. Comput Biol Med.

[31] Hadley C, Awan OA, Zoarski GH (2010). Biomechanics of vertebral bone augmentation. Neuroimaging Clin N Am.

[32] Ayturk UM, Garcia JJ, Puttlitz CM (2010). The micromechanical role of the annulus fibrosus components under physiological loading of the lumbar spine. J Biomech Eng.

[33] Grauer JN, Biyani A, Faizan A, Kiapour A, Sairyo K, Ivanov A, Ebraheim NA, Patel T, Goel VK (2006). Biomechanics of two-level Charité artificial disc placement in comparison to fusion plus single-level disc placement combination. Spine J Off J N Am Spine Soc.

[34] Yoganandan N, Kumaresan S, Pintar FA (2000). Geometric and mechanical properties of human cervical spine ligaments. J Biomech Eng.

[35] Shirazi-Adl A, Ahmed AM, Shrivastava SC (1986). Mechanical response of a lumbar motion segment in axial torque alone and combined with compression. Spine.

[36] Lu T, Lu Y (2019). Comparison of biomechanical performance among posterolateral fusion and transforaminal, extreme, and oblique lumbar interbody fusion: a finite element analysis. World Neurosurg.

[37] Chen YL, Lai OJ, Wang Y, Ma WH, Chen QX (2020). The biomechanical study of a modified lumbar interbody fusion-crenel lateral interbody fusion (CLIF): a three-dimensional finite-element analysis. Comput Methods Biomech Biomed Eng.

[38] Boden SD, Davis DO, Dina TS, Patronas NJ, Wiesel SW (1990). Abnormal magnetic-resonance scans of the lumbar spine in asymptomatic subjects. A prospective investigation. J Bone Joint Surg Am.

[39] Elfering A, Semmer N, Birkhofer D, Zanetti M, Hodler J, Boos N (2002). Risk factors for lumbar disc degeneration: a 5-year prospective MRI study in asymptomatic individuals. Spine.

[40] Patwardhan AG, Havey RM, Meade KP, Lee B, Dunlap B (1999). A follower load increases the load-carrying capacity of the lumbar spine in compression. Spine.

[41] Li QY, Kim HJ, Son J, Kang KT, Chang BS, Lee CK, Seok HS, Yeom JS (2017). Biomechanical analysis of lumbar decompression surgery in relation to degenerative changes in the lumbar spine—validated finite element analysis. Comput Biol Med.

[42] Masni A, Tanaka M (2018). Biomechanical investigation on the influence of the regional material degeneration of an intervertebral disc in a lower lumbar spinal unit: a finite element study. Comput Biol Med.

[43] Schmidt H, Heuer F, Simon U, Kettler A, Rohlmann A, Claes L, Wilke HJ (2006). Application of a new calibration method for a three-dimensional finite element model of a human lumbar annulus fibrosus. Clin Biomech (Bristol, Avon).

[44] Schmidt H, Heuer F, Drumm J, Klezl Z, Claes L, Wilke HJ (2007). Application of a calibration method provides more realistic results for a finite element model of a lumbar spinal segment. Clin Biomech (Bristol, Avon).

[45] Renner SM, Natarajan RN, Patwardhan AG, Havey RM, Voronov LI, Guo BY, Andersson GB, An HS (2007). Novel model to analyze the effect of a large compressive follower pre-load on range of motions in a lumbar spine. J Biomech.

[46] Oxland TR, Lund T (2000). Biomechanics of stand-alone cages and cages in combination with posterior fixation: a literature review. Eur Spine J Off Publ Eur Spine Soc Eur Spinal Deform Soc Eur Sect Cerv Spine Res Soc.

[47] Soriano-Baron H, Newcomb A, Malhotra D, Martinez Del Campo E, Palma AE, Theodore N, Crawford NR, Kelly BP, Kaibara T (2019). Biomechanical effects of an oblique lumbar PEEK cage and posterior augmentation. World Neurosurg.

[48] Lai O, Chen Y, Chen Q, Hu Y, Ma W (2021). Cadaveric biomechanical analysis of multilevel lateral lumbar interbody fusion with and without supplemental instrumentation. BMC Musculoskelet Disord.

[49] Wang B, Hua W, Ke W, Lu S, Li X, Zeng X, Yang C (2019). Biomechanical evaluation of transforaminal lumbar interbody fusion and oblique lumbar interbody fusion on the adjacent segment: a finite element analysis. World Neurosurg.

[50] Tian L, Tang N, Ngai T, Wu C, Ruan Y, Huang L, Qin L (2019). Hybrid fracture fixation systems developed for orthopaedic applications: a general review. J Orthop Transl.

[51] Salvatore G, Berton A, Giambini H, Ciuffreda M, Florio P, Longo UG, Denaro V, Thoreson A, An KN (2018). Biomechanical effects of metastasis in the osteoporotic lumbar spine: a finite element analysis. BMC Musculoskelet Disord.

[52] Wang T, Zhao Y, Cai Z, Wang W, Xia Y, Zheng G, Liang Y, Wang Y (2019). Effect of osteoporosis on internal fixation after spinal osteotomy: a finite element analysis. Clin Biomech (Bristol, Avon).

[53] Nayak AN, Gutierrez S, Billys JB, Santoni BG, Castellvi AE (2013). Biomechanics of lateral plate and pedicle screw constructs in lumbar spines instrumented at two levels with laterally placed interbody cages. Spine J Off J N Am Spine Soc.

[54] Liu JM, Zhang Y, Zhou Y, Chen XY, Huang SH, Hua ZK, Liu ZL (2017). The effect of screw tunnels on the biomechanical stability of vertebral body after pedicle screws removal: a finite element analysis. Int Orthop.

[55] Wáng YXJ, Deng M, He LC, Che-Nordin N, Santiago FR (2018). Osteoporotic vertebral endplate and cortex fractures: a pictorial review. J Orthop Transl.

[56] Galbusera F, Mietsch A, Schmidt H, Wilke HJ, Neidlinger-Wilke C (2013). Effect of intervertebral disc degeneration on disc cell viability: a numerical investigation. Comput Methods Biomech Biomed Eng.

